# Drivers of Acceptance of COVID-19 Proximity Tracing Apps in Switzerland: Panel Survey Analysis

**DOI:** 10.2196/25701

**Published:** 2021-01-06

**Authors:** Viktor von Wyl, Marc Höglinger, Chloé Sieber, Marco Kaufmann, André Moser, Miquel Serra-Burriel, Tala Ballouz, Dominik Menges, Anja Frei, Milo Alan Puhan

**Affiliations:** 1 Epdemiology, Biostatistics & Prevention Institute University of Zurich Zürich Switzerland; 2 Institute for Implementation Science in Health Care University of Zurich Zürich Switzerland; 3 Winterthur Institute of Health Economics Zurich University of Applied Sciences Winterthur Switzerland

**Keywords:** COVID-19, SARS-CoV-2, digital proximity tracing, digital contact tracing, mHealth, tracing, compliance, acceptance, uptake, usability, communication

## Abstract

**Background:**

Digital proximity tracing apps have been released to mitigate the transmission of SARS-CoV-2, the virus known to cause COVID-19. However, it remains unclear how the acceptance and uptake of these apps can be improved.

**Objective:**

This study aimed to investigate the coverage of the SwissCovid app and the reasons for its nonuse in Switzerland during a period of increasing incidence of COVID-19 cases.

**Methods:**

We collected data between September 28 and October 8, 2020, via a nationwide online panel survey (COVID-19 Social Monitor, N=1511). We examined sociodemographic and behavioral factors associated with app use by using multivariable logistic regression, whereas reasons for app nonuse were analyzed descriptively.

**Results:**

Overall, 46.5% (703/1511) of the survey participants reported they used the SwissCovid app, which was an increase from 43.9% (662/1508) reported in the previous study wave conducted in July 2020. A higher monthly household income (ie, income >CHF 10,000 or >US $11,000 vs income ≤CHF 6000 or <US $6600 [reference]: odds ratio [OR] 1.92, 95% CI 1.40-2.64), more frequent internet use (ie, daily [reference] vs less than weekly: OR 0.37, 95% CI 0.16-0.85), better adherence to recommendations for wearing masks (ie, always or most of the time [reference] vs rarely or never: OR 0.28, 95% CI 0.15-0.52), and nonsmoker status (OR 1.32, 95% CI 1.01-1.71) were associated with an increased likelihood for app uptake. Citizenship status (ie, non-Swiss citizenship vs. Swiss [reference]: OR 0.61, 95% CI 0.43-0.87), and language region (French vs Swiss German [reference]: OR 0.61, 95% CI 0.46-0.80) were associated with a lower likelihood for app uptake. Further analysis in a randomly selected subsample (n=712) with more detailed information showed that higher levels of trust in government and health authorities were also associated with a higher likelihood for app uptake (ie, high vs low [reference] trust: OR 3.13, 95% CI 1.58-6.22). The most frequent reasons for app nonuse were lack of perceived benefit of using the app (297/808, 36.8%), followed by the lack of a compatible phone (184/808, 22.8%), and privacy concerns (181/808, 22.4%).

**Conclusions:**

Eliminating technical hurdles and communicating the benefits of digital proximity tracing apps are crucial to promote further uptake and adherence of such apps and, ultimately, enhance their effectiveness to aid pandemic mitigation strategies.

## Introduction

### Background

Safe and effective vaccines against SARS-CoV-2, the causative agent of COVID-19, are not largely available in most countries. Therefore, global and national health authorities continue to rely on nonpharmaceutical interventions in their fight against the ongoing COVID-19 pandemic. Cornerstones of pandemic mitigation measures include testing, tracing, isolation, and quarantine [1]. Digital proximity tracing (DPT) apps are expected to further enhance conventional mitigation measures, and classic, interview-based contact tracing in particular. These apps constitute a novel, still largely untested health technology that anonymously records the user’s proximity contacts, that is, other app users who were within a prespecified radius for a certain amount of time [2]. In case the app user tests positive for COVID-19, they can notify their proximity contacts in an anonymous manner through such DPT apps.

The rationales for using DPT apps as pandemic mitigation tools are based on a modelling study, which suggests that DPT alone has the ability to stop the spread of the COVID-19 pandemic [3,4]. Classic contact tracing is labor- and time-consuming, and exposed contacts can sometimes only be reached and notified with substantial time lags [5]. By comparison, DPT can lead to faster notification and earlier self-quarantine of exposed contacts [3,6]. In addition, DPT has a wider reach than classic contact tracing, as it also includes exposed contacts that the infected person may not know by name (eg, chance encounters in a public space). However, the modelling studies further suggest that these expected effects of digital contact tracing depend on several assumptions. Specifically, a considerable proportion of the population must use the DPT app (eg, 60% and more if no other mitigation measures are implemented), the turnaround time of test results and digital notification of exposed contacts must be within 1-2 days and notified contacts should enter self-quarantine immediately [3,7].

### How the Swiss DPT App (SwissCovid) Works

The Swiss DPT app, officially named “SwissCovid,” follows the blueprint of decentralized, privacy-preserving proximity tracing (DP-3T). Detailed explanations of the DP-3T design can be found elsewhere [2,6]. The DP-3T app architecture has also become the basis for national DPT apps in countries such as Italy or Germany, and it has gained the support of Apple and Google, who provide application programming interfaces to support the app’s functionality [8].

The SwissCovid app was publicly released on June 25, 2020 [9]. Similar to other DP-3T–inspired apps, smartphones with the SwissCovid app installed will send and receive Bluetooth Low Energy signals to and from other smartphones that also have the same app installed. Ephemeral, nonidentifiable keys are exchanged and stored locally on smartphones. As Bluetooth signals weaken with increasing distance between devices, signal attenuation can be employed to determine whether another phone or device was in close proximity (eg, <1.5 meters) and, if so, for how long. If any app user tests positive for COVID-19, this person will be issued an activation code (CovidCode) that should be entered into the SwissCovid app. By doing so, the user releases their ephemeral keys, which are then uploaded onto a central server system.

Smartphones with DP-3T–based apps regularly connect to this central server. The uploaded keys of users with known infection are downloaded by all smartphones using the SwissCovid app, and the smartphone user’s locally stored encounter-history (ie, the list of exchanged keys) will be searched for matches with keys of infected users. If matches fulfilling the criteria for a close proximity encounter (<1.5 meters over at least 15 minutes) are found, the smartphone owner will be notified and advised to call an Infoline for further assistance. Users who receive such a notification are also advised to enter self-quarantine and undergo testing for COVID-19.

Thus, the effect of proximity tracing on pandemic containment is mediated by users being notified about possible exposure risks as soon as possible and entering quarantine to break further transmission chains (ie, by being “one step ahead”). However, emerging data from Switzerland indicate that procedural aspects (eg, speed of laboratory test results and delivery of CovidCodes) and user behavior (eg, the period before contacting the infoline after receiving an app notification of contact with another app user who has tested positive) have an influence on the performance of the DPT app notification cascade [10,11]. For example, frequent delays in issuing activation codes for app users who have tested positive for COVID-19 also delayed notification of exposed contacts (monitored in [12]).

### Study Aims

DPT technologies have been developed and implemented with very limited real-life testing [1,13]. It currently remains unclear whether, and to what extent, assumptions stated by the modelling analyses are achievable under real-world conditions and whether these technologies can ultimately have a significant impact on the effectiveness of pandemic mitigation strategies [13,14].

Therefore, in this study, we aimed to investigate and synthesize to what extent some of the conditions for DPT functioning (namely, broad app uptake) were fulfilled during the first 3 months after the release of the SwissCovid app in Switzerland. Our analyses addressed 3 main research questions: (1) Which sociodemographic and health-related factors are associated with use of the SwissCovid app? (2) What are the most prominent concerns for nonuse of the SwissCovid app? (3) What is known about the adherence of app users with the recommended procedures in case of an app notification indicating proximity contact with another app user who has tested positive for COVID-19? To answer these questions, we analyzed data collected from a web-based nationwide, survey panel, complemented by publicly available data.

## Methods

### Data Source

This study was based on survey data collected from the Swiss COVID-19 Social Monitor project [15], a cohort study of participants randomly selected from an existing online panel population. A weighted sample from the panel, stratified based on age, gender, and language region, was used in order to make the sample representative of the Swiss population. Participants of this cohort receive an invitation every 2-6 weeks to complete a survey on various COVID-19 related topics. The survey was started on March 30, 2020; thus far, 10 study waves have been conducted, each with an average response from 1500-1700 persons from across Switzerland. All datasets generated and/or analyzed during this study are available from the corresponding author on reasonable request.

The Swiss COVID-19 Social Monitor project collects information on sociodemographic features, comorbidities, and implementation of preventive measures related to COVID-19. In addition, 3 standardized questions were introduced to gather information about the use of the Swiss DPT app (SwissCovid; see Table S1 in Multimedia Appendix 1). The questions were jointly developed by study investigators, epidemiologists, and infectious disease experts. The standardized SwissCovid app-related questions were first introduced in Wave 8 and subsequently used in Waves 9 and 10.

The primary data source for these analyses was Wave 10 of the Swiss COVID-19 Social Monitor project (September 28 to October 8, 2020), which yielded responses from 1511 participants. Additional data on media use and trust in government, health authorities, or science, were collected for a randomly selected subsample in Wave 10 (n=712; Table S2 in Multimedia Appendix 1). Furthermore, data from 1299 participants were collected from Wave 8 (July 13-20, 2020) as well as Wave 10, which were used for analyzing within-person-changes in SwissCovid app use over time and reasons for app nonuse over time. Data from Waves 8, 9 (August 17-25, 2020), and 10 were used to evaluate user responses to app notifications.

### Context of the Pandemic Situation

The observation period for this study started from app release on June 25, 2020, to approximately 3 months thereafter. By early October 2020, the app was downloaded 2.4 million times, and the number of active users was relatively stable at 1.6 million [12]. Active users were counted as the daily number of app dummy requests sent to the proximity tracing system, which tends to underestimate the real number of users [16]. Compared with Switzerland’s population size of 8.6 million persons of all age groups (6.6 million in the age group between 18 and 79 years), the number of active app users corresponds to a population coverage of approximately 19% (24.2% among those aged 18-79 years).

In hindsight, the time period of this survey marked the starting point for large increases in the incidence of COVID-19 cases in Switzerland. A total of 8114 new COVID-19 cases (based on positive polymerase chain reaction tests) were reported during the study period, that is, from September 28, 2020, to October 8, 2020. By contrast, the number of new COVID-19 cases was considerably lower in the preceding 11-day period (ie, 3644 cases during September 17-27, 2020) [17].

### Ethics Statement

For the COVID-19 Social Monitor project, the Ethics Committee of the Canton of Zurich confirmed that it does not fall under the Swiss Human Research Law (BASEC-Nr. Req-2020-00323). Therefore, informed consent from participants was not needed.

### Measures

To study the uptake of the SwissCovid app, users and nonusers were compared by age (in 10-year categories), gender, partnership status, having children, citizenship, language region, education status, employment status, household income, smoking status, presence of self-reported comorbidities (eg, respiratory diseases, cardiovascular diseases, stroke, hypertension, diabetes, and cancer), application of preventive measures (eg, wearing masks and staying at home except for essential tasks), frequency of internet use, trust in government and health authorities, and trust in science. Individuals who reported they used the app permanently or who turned it off only occasionally were considered “app users” for the purpose of this study. Other individuals who reported not using the app (either with or without an intention to do so later) were considered “app nonusers.”

### Statistical Analyses

#### Factors Associated With App Uptake

Descriptive analyses were performed by summarizing continuous data as medians (interquartile ranges) and categorical data as percentages. Changes in app use status between Waves 8 and 10 were also analyzed descriptively among participants who contributed to both waves.

To investigate factors associated with app use, multivariable logistic regression models were constructed using the abovementioned measures as variables of interest. Age, gender, and comorbidity status were included as a priori fixed co-variables in all models; the remaining variables, including an a priori defined interaction term for age and gender, were added incrementally and retained if the Akaike Information Coefficient decreased by 2 points or more upon variable addition [18,19]. Further logistic regression analyses on the association between app use and media use and trust in government or science were performed for the subset of participants for whom this information was available. Results from regression analyses are reported as odds ratio (OR) with 95% confidence intervals.

#### Investigation of Reasons for App Nonuse

Reasons for nonuse of the SwissCovid app were further explored descriptively represented as n (%) based on the answer options provided, as well as an open answer field for describing other reasons. The analysis was limited to one primary reason for each participant. Sociodemographic and other characteristics as listed above were compared descriptively across the 3 most frequent reasons for app nonuse, as well as a fourth group subsuming all other reasons. All analyses were performed using Stata version 13 (Stata Corp).

## Results

### Sample Characteristics

The Wave 10 survey yielded 1511 responses; participant characteristics are shown in Table 1. The median age of survey participants was 48 years, and 48.8% (738/1511) were female. Almost two-thirds (975/1511, 64.5%) of the participants lived in the German language region, 22.1% (334/1511) lived in the French language region, and 13.4% (202/1511) lived in the Italian language regions. Furthermore, 46.5% (703/1511) of the participants reported to have the app installed, of which 7.7% (116/1511) occasionally switched it off. By comparison, app installation coverage was 43.9% (662/1508) in Wave 8 (data not shown). Among the 1299 respondents participating in both Waves 8 and 10, only 75 of 733 (10.2%) app nonusers from Wave 8 had the app installed by Wave 10 (data not shown). However, 5.3% (30/566) of the app users from Wave 8 had uninstalled the app by Wave 10.

**Table 1:** Study populations of users and nonusers of the SwissCovid app, deployed in Switzerland as a mitigation measure for the COVID-19 pandemic.

Characteristic		Value		
		Social Monitor Project (N=1511)	App nonuse (n=808)	App use (n=703)
Age, median (IQR)		48 (34, 59)	49 (35, 58)	46 (34, 59)
Gender, female, n (%)		738 (48.8)	389 (48.1)	349 (49.6)
**Partnership status, n (%)**				
	No partner	440 (29.1)	246 (30.4)	194 (27.6)
	Living with partner	951 (62.9)	490 (60.6)	461 (65.6)
	Not living with partner	120 (7.9)	72 (8.9)	48 (6.8)
Has children, yes, n (%)		163 (10.8)	92 (11.4)	71 (10.1)
**Citizenship status, n (%) **				
	Swiss	1220 (80.7)	624 (77.2)	596 (84.8)
	Swiss and other	129 (8.5)	83 (10.3)	46 (6.5)
	Non-Swiss	162 (10.7)	101 (12.5)	61 (8.7)
**Language region, n (%)**				
	German	975 (64.5)	494 (61.1)	481 (68.4)
	French	334 (22.1)	200 (24.8)	134 (19.1)
	Ticino	202 (13.4)	114 (14.1)	88 (12.5)
**Education, n (%)**				
	Only mandatory schooling	93 (6.2)	60 (7.4)	33 (4.7)
	Completed professional education	728 (48.2)	406 (50.2)	322 (45.8)
	University or university of applied sciences	690 (45.7)	342 (42.3)	348 (49.5)
Currently employed, n (%)		1066 (70.5)	563 (69.7)	503 (71.6)
**Monthly household income, n (%)**				
	≤CHF 6000 (US $6600)	397 (26.3)	246 (30.4)	151 (21.5)
	CHF 6000-10,000 (US $6600-11,000)	491 (32.5)	261 (32.3)	230 (32.7)
	>CHF 10,000 (US $11,000)	343 (22.7)	146 (18.1)	197 (28)
	No answer	280 (18.5)	155 (19.2)	125 (17.8)
Smoker, yes, n (%)		313 (20.7)	188 (23.3)	125 (17.8)
Self-reported chronic illness^a^, n (%)		378 (25)	197 (24.4)	181 (25.7)
**Use of protective masks, n (%)**				
	Always or most of the time	962 (63.7)	494 (61.1)	468 (66.6)
	Sometimes	484 (32)	264 (32.7)	220 (31.3)
	Rarely or never	65 (4.3)	50 (6.2)	15 (2.1)
**Staying at home except for essential tasks, n (%)**				
	Always or most of the time	409 (27.1)	224 (27.7)	185 (26.3)
	Sometimes	622 (41.2)	316 (39.1)	306 (43.5)
	Rarely or never	480 (31.8)	268 (33.2)	212 (30.2)
**Frequency of internet use, n (%)**				
	Once daily or several times a day	1329 (88)	685 (84.8)	644 (91.6)
	Once weekly or several days per week	150 (9.9)	99 (12.3)	51 (7.3)
	Never or less than once weekly	32 (2.1)	24 (3)	8 (1.1)
**Trust in government^b^, n (%) **				
	Little	60/712 (8.4)	47/375 (12.5)	13/337 (3.9)
	Somewhat	163/712 (22.9)	102/375 (27.2)	61/337 (18.1)
	Large	489/712 (68.7)	226/375 (60.3)	263/337 (78)
**Trust in science^b^, n (%)**				
	Little	58/710 (8.2)	42/374 (11.2)	16/336 (4.8)
	Somewhat	207/710 (29.2)	129/374 (34.5)	78/336 (23.2)
	Large	445/710 (62.7)	203/374 (54.3)	242/336 (72)
**SwissCovid app use, n (%)** ** **				
	App user	587 (38.8)	N/A^c^	N/A
	App user, occasionally switching off the app	116 (7.7)	N/A	N/A
	Intends to use the app	53 (3.5)	N/A	N/A
	Has uninstalled app	66 (4.4)	N/A	N/A
	Not using the app	689 (45.6)	N/A	N/A

^a^Presence of chronic illness was defined based on self-reporting of at least one of the following conditions: asthma, chronic obstructive pulmonary disease, diabetes, hypertension, cardiovascular disease, stroke, and cancer.

^b^Data only available in a randomly selected split-sample, including 47.1% (712/1511) of the full study population.

^c^N/A: not applicable.

### Factors Associated With App Uptake

Multivariable logistic regression analyses revealed that several factors were associated with app uptake (see Table 2). Analysis of the full study sample showed that citizenship status (Swiss and second citizenship: OR 0.58, 95% CI 0.40-0.86; non–Swiss citizenship: OR 0.61, 95% CI 0.43-0.87 vs Swiss citizenship only), and language region (French-speaking region: OR 0.61, 95% CI 0.46-0.80; Italian-speaking region: OR 0.78, 95% CI 0.57-1.08 vs German-speaking region) were associated with lower app uptake.

By contrast, a higher monthly household income (OR 1.92, 95% CI 1.40-2.64 for an income >CHF 10,000 [US $11,000] vs income ≤CHF 6000 [US $6600]), more frequent internet use (daily [reference] vs less than weekly: OR 0.37, 95% CI 0.16-0.85), better adherence to mask-wearing recommendations (always or most of the time [reference] vs rarely or never OR 0.28, 95% CI 0.15-0.52), and nonsmoker status (OR 1.32, 95% CI 1.01-1.71) were associated with increased app uptake.

The same model was also applied to the random subsample (see Table 2), which provided additional information on trust in government and science (n=712). Of note, ORs of variables included in both multivariable models (ie, full and subsample) were not altered substantially, but CIs became wider due to the lower sample size. Furthermore, increasing levels of trust in government and health authorities were also associated with a higher likelihood of app uptake (OR 3.13, 95% CI 1.58-6.22 for high vs low [reference] trust), whereas the inclusion of trust in science did not improve the multivariable model fit.

**Table 2:** Results of a multivariable logistic regression analysis investigating factors associated with the use of the SwissCovid app

Characteristic		Value, odds ratio (95% CIs)		
		Univariable; full sample (N=1511)	Multivariable; full sample (N=1511)	Multivariable; random subsample interviewed on trust in government and science (n=712)
Age (per 10 years)		1 (0.99; 1.01)	0.99 (0.92, 1.06)	1.09 (0.98, 1.22)
Female Gender (vs male)		1.06 (0.87, 1.30)	1.10 (0.89, 1.36)	0.94 (0.68, 1.30)
**Partnership status**				
	No partner	ref^a^	N/A^b^	N/A
	Living with partner	1.19 (0.95, 1.50)	N/A	N/A
	Not living with partner	0.85 (0.56, 1.27)	N/A	N/A
Has children (vs not)		0.87 (0.63, 1.21)	N/A	N/A
**Citizenship status**				
	Swiss	ref	ref	ref
	Swiss and other	0.58 (0.40, 0.85)	0.58 (0.40, 0.86)	0.52 (0.28, 0.96)
	Non-Swiss	0.63 (0.45, 0.89)	0.61 (0.43, 0.87)	0.68 (0.39, 1.20)
**Language region **				
	German	ref	ref	ref
	French	0.69 (0.53, 0.89)	0.61 (0.46, 0.80)	0.56 (0.37, 0.84)
	Ticino	0.79 (0.58, 1.08)	0.78 (0.57, 1.08)	0.90 (0.54, 1.51)
**Education **				
	Only mandatory schooling	ref	ref	ref
	Completed professional education	1.44 (0.92, 2.26)	1.32 (0.83, 2.12)	1.23 (0.57, 2.63)
	University or university of applied sciences	1.85 (1.18, 2.90)	1.50 (0.94, 2.42)	1.58 (0.73, 3.45)
Currently employed (vs unemployed)		0.91 (0.73, 1.14)	N/A	N/A
**Monthly household income **				
	≤CHF 6000 (US $6600)	ref	ref	ref
	CHF 6000-10,000 (US $6600-11,000)	1.44 (1.10, 1.88)	1.29 (0.97, 1.71)	1.14 (0.75, 1.74)
	>CHF 10,000 (US $11,000)	2.20 (1.64, 2.95)	1.92 (1.40, 2.64)	1.53 (0.94, 2.48)
	No answer	1.31 (0.96, 1.79)	1.18 (0.85, 1.63)	1.06 (0.66, 1.71)
Nonsmoker (vs smoker)		1.40 (1.09, 1.81)	1.32 (1.01, 1.71)	1.51 (1.02, 2.25)
Self-reported chronic illness^c^ (vs none)		1.08 (0.85, 1.36)	1.11 (0.87, 1.43)	0.88 (0.61, 1.27)
**Use of protective masks **				
	Always or most of the time	ref	ref	ref
	Sometimes	0.88 (0.71, 1.10)	0.75 (0.60, 0.96)	0.77 (0.54, 1.10)
	Rarely or never	0.32 (0.18, 0.57)	0.28 (0.15, 0.52)	0.32 (0.12, 0.86)
**Staying at home except for essential tasks**				
	Always or most of the time	ref	N/A	N/A
	Sometimes	1.17 (0.91, 1.51)	N/A	N/A
	Rarely or never	0.96 (0.73, 1.25)	N/A	N/A
**Frequency of internet use**				
	Once daily or several times a day	ref	ref	ref
	Once weekly or several days per week	0.55 (0.38, 0.78)	0.55 (0.38, 0.80)	0.59 (0.35, 1.00)
	Never or less than once weekly	0.35 (0.16, 0.79)	0.37 (0.16, 0.85)	0.32 (0.11, 0.95)
**Trust in government or health authorities^d^**				
	Little	ref	N/A	ref
	Somewhat	2.16 (1.08, 4.32)	N/A	1.71 (0.82, 3.58)
	Large	4.21 (2.22, 7.97)	N/A	3.13 (1.58, 6.22)
**Trust in science^d^**				
	Little	ref	N/A	N/A
	Somewhat	1.59 (0.84, 3.01)	N/A	N/A
	Large	3.13 (1.71, 5.73)	N/A	N/A

^a^ref: reference value.

^b^N/A: data not applicable or not included because it did not improve model fit

^c^Presence of chronic illness was defined based on self-reporting of at least one of the following conditions: asthma, chronic obstructive pulmonary disease, diabetes, hypertension, cardiovascular disease, stroke, and cancer.

^d^Data only available in a randomly selected split-sample, including 47% (712/1511) of the full study population.

### Reasons for App Nonuse

The responses of participants who reported not having used the app (808/1511) were analyzed further with respect to the reasons for app nonuse (Table 3). This group included both users who stated that they intended to use the app and those who did not intend to use it. Overall, the most important reasons for not installing the app were a perceived lack of usefulness of the app (297/808, 36.8%), followed by not having a suitable smartphone or operating system (184/808, 22.8%), and concerns about privacy (181/808, 22.4%). Other reasons (amounting to 18%) included lack of knowledge about the app, doubts about technological reliability, and concerns about excessive battery usage, among other reasons.

When compared to responses from Wave 8, the proportion of app nonusers (846/1508, 56.1%) who reported a perceived lack of app usefulness (228/846, 27%) was considerably lower. Moreover, the differences between waves for the other reasons of app nonuse (ie, not having the right phone: 221/846, 26.1%; privacy concerns: 202/846, 23.9%; and other reasons: 195/846, 23%; data not shown) were less pronounced.

As shown in Table 3, the distribution of reasons for app nonuse also varied with participants’ intentions for using the app later (ie, maybe, no, or already uninstalled the app). While the lack of perceived benefits was the dominant reason for not installing the app (262/689, 38%) and having uninstalled (20/66, 30.3%) it, 34% (18/53) of the participants who intended to install the app at a later time point reported not having a compatible smartphone. Furthermore, it is noteworthy that excessive battery consumption also appeared to be an important reason for uninstalling the app (11/66, 16.7%).

The descriptive comparison of sociodemographic and other characteristics across the 3 major reasons for app nonuse (and a fourth category subsuming all other reasons; see Table 4) suggests that some reasons may be more prevalent in specific subgroups. The subpopulation citing problems with installing the app (“not the right phone”) was the oldest (median age, 57.5 years), had the highest burden of chronic comorbidities (61/184, 33.2%), and tended to have high trust in government (large trust category: 69/89, 77.5%) and science (large trust category: 61/89, 68.5%) compared with the other subgroups. By contrast, those reporting privacy concerns for app nonuse were younger (median age: 44 years), more frequently living in the French-speaking part of Switzerland (65/181, 35.9%), and generally had less trust in the government (large trust category: 35/80, 43.8%) or science (large trust category: 30/80, 37.5%). No specific patterns were observed for the demographics of subpopulations reporting the remaining 2 reasons (ie, “not useful” and “other reasons”).

**Table 3:** Reasons for nonuse of the SwissCovid app.

Reason	Value, n (%)			
	May install app later (n=53)	App not installed (n=689)	Uninstalled app (n=66)	All (n=808)
Perceived as not useful	15 (28.3)	262 (38)	20 (30.3)	297 (36.8)
Not the right phone	18 (34)	158 (22.9)	8 (12.1)	184 (22.8)
Concerned about privacy	8 (15.1)	164 (23.8)	9 (13.6)	181 (22.4)
Don't know the app	2 (3.8)	25 (3.6)	0 (0)	27 (3.3)
Technical doubts about reliability, maturity	1 (1.9)	20 (2.9)	4 (6.1)	25 (3.1)
Concerned about battery use	1 (1.9)	8 (1.2)	11 (16.7)	20 (2.5)
Don't believe in seriousness of Corona; lack of trust in government	0 (0)	9 (1.3)	1 (1.5)	10 (1.2)
Inertia, not had the time yet	5 (9.4)	2 (0.3)	0 (0)	7 (0.9)
Opposed out of principle, no specific reason	0 (0)	7 (1)	0 (0)	7 (0.9)
Don't want Bluetooth permanently on	0 (0)	3 (0.4)	2 (3)	5 (0.6)
Worried about consequences/quarantine	0 (0)	2 (0.3)	2 (3)	4 (0.5)
Currently outside of Switzerland	1 (1.9)	3 (0.4)	0 (0)	4 (0.5)
Would have to turn off app at work	0 (0)	2 (0.3)	1 (1.5)	3 (0.4)
Would feel stressed/scared by app use	0 (0)	2 (0.3)	0 (0)	2 (0.2)
Already protecting themselves, rarely leave the house	0 (0)	1 (0.1)	0 (0)	1 (0.1)

**Table 4:** Sociodemographic characteristics of SwissCovid app nonusers, stratified by the reason for app nonuse (3 most frequent reasons and “other”).

Characteristic		Value				
		Not the right phone (n=184)	Privacy concerns (n=181)	Not useful (n=297)	Other reason (n=146)	
Age, median (IQR)		57.5 (44.5, 67)	44 (35, 54)	46 (31, 57)	44 (31, 57)	
Gender, female, n (%)		95 (51.6)	102 (56.4)	120 (40.4)	72 (49.3)	
**Partnership status, n (%)**						
	No partner	45 (24.5)	58 (32)	100 (33.7)	43 (29.5)	
	Living with partner	123 (66.8)	106 (58.6)	172 (57.9)	89 (61)	
	Not living with partner	16 (8.7)	17 (9.4)	25 (8.4)	14 (9.6)	
Has children		15 (8.2)	21 (11.6)	27 (9.1)	29 (19.9)	
**Citizenship status, n (%) **						
	Swiss	152 (82.6)	132 (72.9)	232 (78.1)	108 (74)	
	Swiss and other	10 (5.4)	21 (11.6)	35 (11.8)	17 (11.6)	
	Non-Swiss	22 (12)	28 (15.5)	30 (10.1)	21 (14.4)	
**Language region, n (%) **						
	German	115 (62.5)	100 (55.2)	188 (63.3)	91 (62.3)	
	French	41 (22.3)	65 (35.9)	64 (21.5)	30 (20.5)	
	Ticino	28 (15.2)	16 (8.8)	45 (15.2)	25 (17.1)	
**Education, n (%) **						
	Only mandatory schooling	16 (8.7)	16 (8.8)	22 (7.4)	6 (4.1)	
	Completed professional education	90 (48.9)	83 (45.9)	157 (52.9)	76 (52.1)	
	University or university of applied sciences	78 (42.4)	82 (45.3)	118 (39.7)	64 (43.8)	
Currently employed, n (%)		97 (52.7)	140 (77.3)	221 (74.4)	105 (71.9)	
**Monthly household income, n (%)**						
	≤CHF 6000 (US $6600)	65 (35.3)	52 (28.7)	87 (29.3)	42 (28.8)	
	CHF 6000-10,000 (US $6600-11,000)	62 (33.7)	48 (26.5)	104 (35)	47 (32.2)	
	>CHF 10,000 (US $11,000)	25 (13.6)	30 (16.6)	61 (20.5)	30 (20.5)	
	No answer	32 (17.4)	51 (28.2)	45 (15.2)	27 (18.5)	
Smoker, n (%)		36 (19.6)	47 (26)	78 (26.3)	27 (18.5)	
Self-reported chronic illness^a^, n (%)		61 (33.2)	43 (23.8)	66 (22.2)	27 (18.5)	
**Use of protective masks, n (%)**						
	Always or most of the time	127 (69)	110 (60.8)	172 (57.9)	85 (58.2)	
	Sometimes	50 (27.2)	62 (34.3)	106 (35.7)	46 (31.5)	
	Rarely or never	7 (3.8)	9 (5)	19 (6.4)	15 (10.3)	
**Staying at home except for essential tasks, n (%)**						
	Always or most of the time	54 (29.3)	48 (26.5)	83 (27.9)	39 (26.7)	
	Sometimes	90 (48.9)	69 (38.1)	108 (36.4)	49 (33.6)	
	Rarely or never	40 (21.7)	64 (35.4)	106 (35.7)	58 (39.7)	
**Frequency of internet use, n (%)**						
	Once daily or several times a day	142 (77.2)	160 (88.4)	257 (86.5)	126 (86.3)	
	Once weekly or several days per week	32 (17.4)	14 (7.7)	36 (12.1)	17 (11.6)	
	Never or less than once weekly	10 (5.4)	7 (3.9)	4 (1.3)	3 (2.1)	
**Trust in government^b^, n (%) **						
	Little	5 (5.6)	16 (20)	12 (8.6)	14 (21.2)	
	Somewhat	15 (16.9)	29 (36.3)	43 (30.7)	15 (22.7)	
	Large	69 (77.5)	35 (43.8)	85 (60.7)	37 (56.1)	
**Trust in science^b^, n (%) **						
	Little	7 (7.9)	13 (16.3)	12 (8.6)	10 (15.2)	
	Somewhat	21 (23.6)	37 (46.3)	47 (33.8)	24 (36.4)	
	Large	61 (68.5)	30 (37.5)	80 (57.6)	32 (48.5)	

^a^Presence of chronic illness was defined based on self-reporting of at least one of the following conditions: asthma, chronic obstructive pulmonary disease, diabetes, hypertension, cardiovascular disease, stroke, and cancer.

^b^Data only available in a randomly selected split-sample, including 50% of the full study population (n=712). New denominators for respective reasons of app nonuse were not right phone (n=89), privacy concerns (n=80), not useful (n=140), and other (n=66).

### SwissCovid App Notifications and User Response

In the 3 survey waves (Waves 8, 9, and 10), a total of 15 participants reported having received an app notification: 2 users in Wave 8 (July), 6 users in Wave 9 (August), and 7 users in Wave 10 (October). Overall, 8 of these 15 (53.3%) users reported to have called the recommended infoline, whereas the remaining 6 users reported not to have undertaken any steps, and 1 user undertook other steps, which were left unspecified.

Since Wave 10, participants were also asked whether they had undergone COVID-19 testing in the past 4 weeks and, if so, what their test results were. Of the 5 users who called the infoline at Wave 10, 2 users reported to have undergone COVID-19 testing, and 1 of them reported to have tested positive for COVID-19.

## Discussion

### Principal Findings

By analyzing information on the use of the SwissCovid app from a longitudinal, web-based, panel survey, we evaluated factors related to the use of the DPT app in Switzerland.

Our data suggested that, 3 months after app release, 46.5% of the survey respondents had downloaded the app (of whom 38.8% had the SwissCovid app permanently activated). This proportion is an overestimation of actual app coverage in the general population and is most likely caused by the above-average affinity for such technologies of online panel participants. Moreover, social desirability might have led to some over-reporting of app use, despite this being an anonymous online survey [20]. In early October 2020, the official number of active app users was estimated at 1.6 million [12], which implies that around 1 in 4 (24.2%) adults residing in Switzerland were actively using the app. A recent modelling study suggests that this proportion of app uptake may, in fact, be sufficient to reduce the number of new infections to “manageable levels” [21].

We also deduced several population characteristics that may influence the uptake of the SwissCovid app. For example, younger age, higher income, or a nonsmoker status were associated with a higher app uptake. In contrast, characteristics such as foreign (non-Swiss) nationality or living in the French- or Italian-speaking regions of Switzerland were associated with a lower app uptake. Furthermore, app uptake was associated with the level of trust placed in the government and health authorities. Following recommended preventive measures and wearing masks, in particular, were also associated with a higher likelihood to use the app, which could imply higher levels of awareness, worry related to the COVID-19 pandemic, or increased health consciousness.

We further investigated participants’ stated reasons for nonuse of the SwissCovid app, which were dominated by technical aspects (ie, not having a suitable smartphone or operating system), privacy concerns, and perceived lack of usefulness. Ignorance or lack of information about the app did not seem to be a relevant reason, as only 3% of the participants cited this as a reason (ie, “don’t know the app”). Privacy concerns as a reason for app nonuse were associated with a lack of trust in the government and health authorities, as well as with a migration background. Participants whose app use was hindered owing to technical reasons seemed to be more trustful in the government but tended to be older. Therefore, streamlining installation processes and establishing compatibility with older phone devices may be worthwhile in order to increase app uptake among this subgroup.

### Comparison With Previous Work

To our knowledge, this is the first study since the SwissCovid app release to systematically investigate DPT app uptake and the reasons for app nonuse in Switzerland. One survey was conducted since the app release in late-June 2020 in Switzerland, comprising 1000 Swiss individuals [22]; however, the data have not been published in detail. This previous study found that 43% of the Swiss population were using or considering using the Swiss proximity tracing app, with higher percentages observed among younger respondents. Our study results show similar proportions of app users.

Overall, our findings also correspond well with other international population surveys of DPT app use, most of which were performed before [23-29], and some after app release [30]. For example, several studies confirmed the finding that higher education status [25], and younger age [23] were associated with a higher willingness to use DPT apps. Of note, these characteristics could reflect the profile of typical early adopters of technology [31]. On the other hand, the observed sociodemographic patterns could also reflect generally higher health- and digital literacy among DPT app users. This alternative explanation raises concerns about the existence of a digital divide [32], in which individual who may benefit the most from the preventive effect of DPT are the least likely to use the app. Nevertheless, as suggested by a separate analysis of the COVID-19 Social Monitor project data, the majority of elderly persons adhered well to other preventive measures such as social distancing [33]. Furthermore, it can also be argued that transmission prevention among younger persons, who are disproportionately affected by the COVID-19 incidence in Switzerland, may also yield a protective effect for older adults.

This study adds to the scarce literature on motivations, as well as technical and nontechnical barriers for app use in settings where apps have already been deployed. Previous studies have utilized models based on psychology such as the health belief model (HBM) [29] and implementation science such as the normalization process theory (NPT) [10] to analyze adoption and nontechnical implementation challenges for DPT apps. Both these models emphasize the importance of a perceived benefit of preventive interventions, with HBM focusing on individuals and NPT focusing on a systems perspective [34]. The lack of perceived benefits was also a major reason for app nonuse in our survey, cited by 37% of all nonusers. Therefore, according to HBM and NPT, communicating usefulness of the app to individual and the society may be key to achieve greater app adoption; this can be achieved by testimonials of users who have had a positive experience with the DPT app. Moreover, optimizing economic incentives or removing existing disincentives for DPT use may further improve the benefit-risk balance [35,36]. In Switzerland, users who received an app notification were eligible for a free COVID-19 test, but quarantine was neither mandatory nor subject to salary compensations (which is currently being reconsidered).

Our observations of technical problems and persistent privacy concerns as reasons for nonuse or uninstallations of the SwissCovid app are consistent with those of a study in Australia [30], which reported similar user complaints. Furthermore, (a lack of) government trust emerged as a strong influencing factor for app usage in our and other surveys [23]. For example, approximately 11% of the respondents of the Australian survey cited government mistrust as a reason for not using the app [30]. In Switzerland, there was an early, rather strong consensus that the DPT app must be issued and managed by the government [37]. Nevertheless, the prevalent and persistent privacy concerns and trust issues remain to be challenging. For example, although the SwissCovid app implements privacy by design, the fact that the app relies on application programming interfaces provided by Google and Apple is sometimes still criticized. One solution to address this challenge could be to establish an independent oversight committee for the management of DPT operations [38]. Such committees would demonstrate transparency and increase public trust that governments will hold their promises, for example, to maintain voluntariness, prevent mission creep, or cease the use of DPT after the end of the pandemic.

Finally, we found evidence that external factors and the overall pandemic context have an effect on benefit and threat perceptions (as postulated by HBM [29]). In Switzerland, COVID-19 cases increased rapidly during the second half of October 2020, and the number of active app users also increased by 200,000 [12]. Similarly, the dominant reasons for app nonuse seem to have evolved in our study. Taken together, these observations suggest that public knowledge, perceptions, and app uptake respond dynamically to the pandemic situation. In line with these suggestions, first reports from vaccine development trials [39] raise hopes for a nearing availability of effective COVID-19 vaccines, which may also impact the public discourse on DPT apps. However, initial vaccination campaigns will likely focus on elderly subpopulations, who are at the greatest risk for a more severe disease course [40]. Given current limitations in vaccine production capacity, herd immunity will remain unachievable in the near future. Therefore, DPT apps will likely continue to play an important role in pandemic mitigation efforts, particularly among younger subpopulations wherein COVID-19 cases are often asymptomatic [40] and DPT app use is comparatively high.

### Strengths and Limitations

Overall, our analysis contributes to the literature by being among the first studies to longitudinally investigate DPT app use patterns in the context of a changing pandemic. A key strength of our study is the availability of data from different survey waves, which allowed us to verify the robustness of our findings. Furthermore, our sample of 1500 participants is based on a random sample and is therefore likely to be quite representative with respect to age, gender, and language region for the Swiss population. However, we cannot fully exclude potential biases such as over-reporting or social desirability bias regarding app use. In addition, the fact that the Social Monitor project sample was drawn from an online panel population might have led to an overestimation of the app use among the general population.

### Conclusions

To summarize, our study findings provide a clearer understanding of the motivations, barriers, and other factors associated with the uptake of DPT apps. Our data points toward complex interactions between motivations, trust, and incentives. Our study also reveals significant research gaps; for example, regarding how to effectively persuade persons with privacy concerns or how to create equitable incentives for app use. Similar studies are needed to evaluate the contribution of DPT on pandemic mitigation efforts, as well as generic, robust research methods to study privacy-preserving health technologies. From a practical perspective, our data suggest that DPT sponsors should scale-up communication efforts to not only build trust and mitigate privacy fears but also reduce technical challenges, as well as simplify onboarding procedures in order to reach a broader population, including persons with low digital or health literacy.

## Appendix

Multimedia Appendix 1 Standardized questions on the use of SwissCovid app based on the COVID-19 Social Monitor project and sociodemographic characteristics of app users who were asked detailed questions about trust in health authorities or science.

## Abbreviations

DPT: digital proximity tracing
DP-3T: decentralized, privacy-preserving proximity tracing
HBM: health belief model
NPT: normalization process theory
OR: odds ratio
