# Supplementary material for: Drivers of Acceptance of COVID-19 Proximity Tracing Apps in Switzerland: Panel Survey Analysis
Source: JMIR Public Health Surveill. 2021 Jan 6;7(1):e25701. doi: 10.2196/25701 (PMC7790736; doi:10.2196/25701)
Supplement: Multimedia Appendix 1 [file publichealth_v7i1e25701_app1.docx]

**Table S1**. Standardized questions on SwissCovid app use in the Social Monitor.

| The SwissCovid App has been launched by the Swiss Federal Office of Public Health to warn smartphone users in case of possible exposure risks. The app records, if a contact has been in close proximity of 1.5m or less for longer than 15 minutes.  If an app user tested positive for the Coronavirus, she or he can anonymously notify other app users, who were in close proximity during the infectious period. | |
| --- | --- |
|  | Are you using the SwissCovid App?   - Yes, permanently - Yes, but sometimes I turn off Bluetooth to pause the SwissCovid App - No, but I am planning to use it - No - Since wave 10: No, I have uninstalled the SwissCovid App |
| Filter If **No or No, but..**: | Why are you currently not using the SwissCovid App?   - I have not heard about the app - I don’t think the app is useful for me - I can’t install the app (e.g., owing to technical difficulties or because I do not own an Android or iOS smartphone) - I fear for my privacy and protection of my data - Other reasons, comment field |
| Filter if **yes or yes, but**: | Were you ever notified by the SwissCovid App that you have been in close proximity to a Corona-positive person?   - No, I have never received a notification - Yes, I called the recommended Infoline SwissCovid - Yes, I undertook other steps; comment field: which? - Yes, but I did not undertake any steps |

**Table S2.** This table compares socio-demographic characteristics of persons between persons who were (“split sample”, N=712) and those who were not included in a random sub-sample (N=799) who were asked more detailed questions about trust in health authorities or science. The survey was conducted in October 2020.

|  | **Split sample (N=712)** | **Not included (N=799)** |
| --- | --- | --- |
| Age, median [IQR] | 49 [36; 59] | 46 [33; 59] |
| Female gender | 340 (47.8%) | 398 (49.8%) |
| Has a partner |  |  |
| No partner | 209 (29.4%) | 231 (28.9%) |
| Living with partner | 451 (63.3%) | 500 (62.6%) |
| Not living with partner | 52 (7.3%) | 68 (8.5%) |
| Has children | 76 (10.7%) | 87 (10.9%) |
| Citizenship |  |  |
| Swiss | 595 (83.6%) | 625 (78.2%) |
| Swiss and other | 54 (7.6%) | 75 (9.4%) |
| Non-Swiss | 63 (8.8%) | 99 (12.4%) |
| Language region |  |  |
| German | 477 (67.0%) | 498 (62.3%) |
| French | 154 (21.6%) | 180 (22.5%) |
| Ticino | 81 (11.4%) | 121 (15.1%) |
| Education |  |  |
| Only mandatory schooling | 37 (5.2%) | 56 (7.0%) |
| Completed professioal education | 343 (48.2%) | 385 (48.2%) |
| University, university of applied sciences | 332 (46.6%) | 358 (44.8%) |
| Currently working | 501 (70.4%) | 565 (70.7%) |
| Monthly household income |  |  |
| ≤CHF 6000 | 183 (25.7%) | 214 (26.8%) |
| CHF 6000 - CHF 10000 | 235 (33.0%) | 256 (32.0%) |
| >CHF 10000 | 156 (21.9%) | 187 (23.4%) |
| No answer | 138 (19.4%) | 142 (17.8%) |
| Smoker | 154 (21.6%) | 159 (19.9%) |
| Self-reported chronic illness** | 181 (25.4%) | 197 (24.7%) |
| Use of protective masks |  |  |
| Always or most of the time | 442 (62.1%) | 520 (65.1%) |
| Sometimes | 242 (34.0%) | 242 (30.3%) |
| Rarely or never | 28 (3.9%) | 37 (4.6%) |
| Staying at home except for esssential tasks |  |  |
| Always or most of the time | 194 (27.2%) | 215 (26.9%) |
| Sometimes | 294 (41.3%) | 328 (41.1%) |
| Rarely or never | 224 (31.5%) | 256 (32.0%) |
| Frequency of internet use |  |  |
| Once daily or several times a day | 616 (86.5%) | 713 (89.2%) |
| Once weekly or several days per week | 78 (11.0%) | 72 (9.0%) |
| Never or less than once weekly | 18 (2.5%) | 14 (1.8%) |
| Questions related to SwissCovid app |  |  |
| Currently using the app | 337 (47.3%) | 366 (45.8%) |
| Not using the app | 375 (52.7%) | 433 (54.2%) |

** Presence of chronic illnesses was defined based on self-report of at least one of the following conditions: asthma, chronic obstructive pulmonary disease (COPD), diabetes, hypertension, cardiovascular disease, stroke, cancer.
